# German Veterinary Medical Association from New York and Vicinity

**Published:** 1894-02

**Authors:** H. Wellner

**Affiliations:** Secretary; New York


### GERMAN VETERINARY MEDICAL ASSOCIATION FROM NEW YORK AND VICINITY.

The regular monthly meeting was held Tuesday evening, January 9th, 1894, at 213 Forsyth Street.

The meeting was called to order by the president, *Dr. L. R. Sattler*, Newark ; upon roll call the following gentlemen responded to their names, viz.: Drs. Turner, Serling, Kuntz, Vogel, Wellner, Patschek and Simmons, Flushing.

The minutes of the previous meeting were read and approved.

Several communications from absent members were read by the secretary, expressing regrets for being unable to attend the meeting. Then the secretary's report was read, which showed the association to be in a flourishing condition numerically, and the roll call showed a large increase. He complained of the lack of interest taken by the members in making the meetings interesting and instructive. He inti mated that association matters were not matters of dollars and cents, or individua- benefit, but were for the advancement of the veterinary profession, and the protection of the public in the State of New York.

The report read by the treasurer showed the association to be in a rather straitened condition financially ; considerable money stands out due the association by members who are in arrears.

*Dr. Patschek* was elected an active member of the association.

*Dr. Sattler* then read a paper on the problems of the veterinarian, and their conduct in their practice. In a discussion in which *Drs. Serling*, *Vogel* and others took part it was concluded to publish an annual report from the association, which should contain all the papers read before this association. The next essayist, *Dr. Vogel*, presented an elaborate paper, the subject being "Vertebrates in health and disease, examined closely from the extreme standpoint of Darwinism. That paper will be continued in the next meeting.

*Dr. Patschek* made a few well chosen remarks on tuberculosis in chickens ; he said the temperature is not higher than in other animals, and he believes that tubercle bacillus can be found in eggs; raw eggs and soft boiled eggs should not be eaten. Then he spoke about the sale of diseased meat and milk, and hoped a proper legislation would regulate this sale. After the subject matter had been discussed by most of the members present, a vote of thanks was tendered the essayists for the able and masterly manner in which they had entertained the meeting.

The next meeting will be held on Tuesday, February 13th, at 3 P. M., at Dr. Sattler's residence, 22 17th Avenue, Newark, N. J.

H. WELLNER, D. V. S.,
*Secretary.*

New York, January 9th, 1894.

